# Portable Dual-Mode Biosensor for Quantitative Determination of *Salmonella* in Lateral Flow Assays Using Machine Learning and Smartphone-Assisted Operation

**DOI:** 10.3390/bios16010057

**Published:** 2026-01-13

**Authors:** Jully Blackshare, Brianna Corman, Bartek Rajwa, J. Paul Robinson, Euiwon Bae

**Affiliations:** 1 Applied Optics Laboratory, School of Mechanical Engineering, Purdue University, West Lafayette, IN 47907, USA; lee4107@purdue.edu; 2 Department of Basic Medical Sciences, Purdue University, West Lafayette, IN 47907, USA; 3 Bindley Bioscience Center, Discovery Park, Purdue University, West Lafayette, IN 47907, USA; 4 Weldon School of Biomedical Engineering, Purdue University, West Lafayette, IN 47907, USA

**Keywords:** lateral flow assay, portable biosensor, photothermal imaging, colorimetric imaging, machine learning, *Salmonella*

## Abstract

Foodborne pathogens remain a major global concern, demanding rapid, accessible, and determination technologies. Conventional methods, such as culture assays and polymerase chain reaction, offer high accuracy but are time-consuming for on-site testing. This study presents a portable, smartphone-assisted dual-mode biosensor that combines colorimetric and photothermal speckle imaging for improved sensitivity in lateral flow assays (LFAs). The prototype device, built using low-cost components ($500), uses a Raspberry Pi for illumination control, image acquisition, and machine learning-based signal analysis. Colorimetric features were derived from normalized RGB intensities, while photothermal responses were obtained from speckle fluctuation metrics during periodic plasmonic heating. Multivariate linear regression, with and without LASSO regularization, was used to predict *Salmonella* concentrations. The comparison revealed that regularization did not significantly improve predictive accuracy indicating that the unregularized linear model is sufficient and that the extracted features are robust without complex penalization. The fused model achieved the best performance (*R*^2^ = 0.91) and consistently predicted concentrations down to a limit of detection (LOD) of 10^4^ CFU/mL, which is one order of magnitude improvement of visual and benchtop measurements from previous work. Blind testing confirmed robustness but also revealed difficulty distinguishing between negative and 10^3^ CFU/mL samples. This work demonstrates a low-cost, field-deployable biosensing platform capable of quantitative pathogen detection, establishing a foundation for the future deployment of smartphone-assisted, machine learning-enabled diagnostic tools for broader monitoring applications.

## 1. Introduction

Foodborne pathogens remain a persistent global threat to public health, affecting nearly 600 million people each year and imposing substantial economic burdens on healthcare systems worldwide [1,2,3,4]. To minimize the risk of outbreaks, contamination must be detected rapidly and accurately before products reach consumers [5,6]. Regulatory agencies such as the U.S. Department of Agriculture (USDA) and the Food and Drug Administration (FDA) have established strict microbial thresholds for food safety, yet existing diagnostic approaches often fail to meet the speed and accessibility requirements for field deployment [7,8].

Conventional methods such as culture-based assays and polymerase chain reaction (PCR) offer high accuracy but are labor-intensive, time-consuming, and require a laboratory setting, making them impractical for rapid screening at points for production or distribution [9,10]. These limitations underscore the need for portable, low-cost biosensing technologies capable of detecting pathogens at low concentrations in real time.

Lateral flow assays (LFAs) have gained widespread adoption in point-of-care diagnostics due to their simplicity, affordability, and rapid delivery results. They are routinely used for detecting a wide range of analytes, from infectious diseases related targets to contaminants in food and water [11,12,13,14]. However, their low sensitivity remains a major obstacle. Commercial LFAs can produce false-negative results when pathogen concentrations fall below their detection limit, particularly in food samples where early detection of contamination is critical [15]. Furthermore, conventional LFAs only provide qualitative results, a binary outcome indicating a test is positive or negative, offering no quantitative information that could assist in assessing contamination severity. Moreover, interference from complex food matrices can suppress signal and reduce assay sensitivity, making conventional LFAs less reliable for pathogen detection in real food samples [16,17,18].

To address this limitation, extensive research has focused on improving LFA sensitivity through either nanoparticle engineering or signal enhancement strategies. While modified tracers, such as platinum or silver nanoparticles, have been shown to enhance optical responses, they require laboratory synthesis and lack the robustness of commercially available gold nanoparticles (AuNPs) [19,20,21,22]. Consequently, alternative enhancement approaches have shifted toward optical and computational sensing methods that improve signal detection without altering assay chemistry [23,24,25,26].

**Figure 1:**
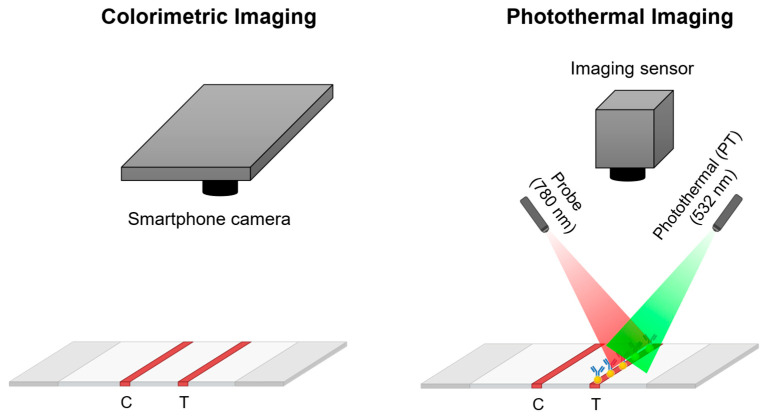
Schematic overview of the two sensing modalities integrated into the dual-mode biosensor. The photothermal imaging diagram depicts the binding event at the test line, where yellow spheres represent gold nanoparticles conjugated with antibodies. The colorimetric mode quantifies optical intensity changes at the test line using RGB image analysis, while the photothermal mode measure speckle fluctuations induced by plasmonic heating under 532 nm excitation.

Two promising optical modalities are colorimetric imaging and photothermal analysis (Figure 1). Colorimetric methods utilize imaging systems, such as smartphone cameras, to capture test lines that may be invisible to the naked eye, followed by image processing or machine learning analysis to quantify the signal. Photothermal sensing, on the other hand, exploits the plasmonic heating properties of AuNPs [27,28]. When irradiated near their resonance wavelength around 520 nm, localized temperature increases can be measured through an infrared camera or photothermal speckle imaging, which tracks refractive-index fluctuations caused by thermal changes [26,28].

In our previous work, we compared these two modalities in a benchtop setup [29]. The results did not show a substantial improvement in detection limit compared to visual inspection. However, they revealed important opportunities for further optimization and integration. This study builds upon those findings, and introduces a portable, smartphone-integrated dual-mode biosensor that combines colorimetric and photothermal sensing into a unified system controlled by a Raspberry Pi microcontroller. The device captures and processes both optical signals, employing machine learning algorithms to quantify test results.

In this work, we investigate whether fusing colorimetric and photothermal modes within a portable platform can enhance sensitivity and reliability beyond what either method might achieve independently. The working hypothesis is that integrating complementary information from each sensing mode will lead to a lower limit of detection (LOD).

The objective of this study is to design, implement, and evaluate a portable dual-mode biosensor for *Salmonella Typhimurium* detection, emphasizing sensitivity improvement, quantitative performance, and on-field usability. The outcome demonstrates the feasibility of achieving a one-order-of-magnitude improvement in detection limit on a smartphone-operable platform suitable for real-time food safety monitoring.

## 2. Materials and Methods

### 2.1. Hardware Architecture

A compact dual-mode biosensing system was developed to integrate optical excitation, image acquisition, and real-time signal processing within a single embedded platform. A Raspberry Pi 5 (Raspberry Pi Ltd., Cambridge, UK) served as the main controller, selected for its processing capability and GPIO interfaces that allow simultaneous control of lighting sources and data acquisition. The Raspberry Pi 5 managed all computation, device communication, and image processing tasks while also serving as a local server to interface with a smartphone application.

A Raspberry Pi camera module 3 (Raspberry Pi Ltd., Cambridge, UK) was selected for image acquisition due to its seamless hardware compatibility and flexible control via the PiCamera2 library [30]. The camera captured images suitable for both colorimetric and photothermal analyses, with adjustable parameters such as exposure time, gain, and frame rate.

Two laser modules were incorporated into the system. A 532 nm 50 mW laser (CivilLaser, Wuhan, China) facilitated photothermal excitation, whereas a 780 nm 30 mW laser (CivilLaser, Wuhan, China) was utilized for speckle generation. The 532 nm laser was transistor-transistor-logic (TTL)-modulated through the Raspberry Pi’s GPIO pins to generate periodic excitation. The 780 nm laser was operated in continuous-wave mode, and its beam diameter was manually adjusted using a dot-focus lens. For colorimetric imaging, two circular white LED rings provided uniform illumination across the testing surface to ensure consistent imaging conditions. Power was supplied through a 5 V DC adapter that distributed current to the laser, camera, and LEDs through a custom-fabricated printed circuit board (PCB) (PCBWay, Shenzhen, China), which was designed using KiCad (KiCad, Geneva, Switzerland).

The Raspberry Pi communicated with a smartphone interface using an HTTP-based protocol, enabling the users to control imaging operations and visualize results in real time. The biosensor communicated with a smartphone interface through an HTTP-based protocol, allowing the users to send operational commands and visualize processed results in real time.

### 2.2. Portable Device Design

**Figure 2:**
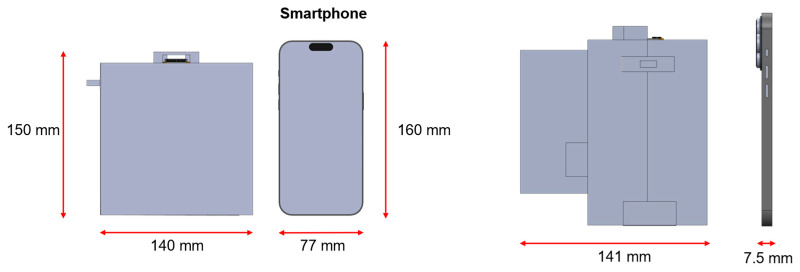
Dimensional comparison of the assembled biosensor with a smartphone, highlighting the compact form factor (150 mm × 140 mm × 140 mm).

A portable enclosure was designed to integrate all hardware components into a compact and field-deployable system. The housing was modeled in SolidWorks 2025 (SOLIDWORKS 3D CAD, Dassault Systèmes, Waltham, MA, USA) and fabricated using an Adventurer 5M 3D printer (Flashforge 3D Technology, Jinhua, China) with PLA filament. The overall dimensions of the assembled device were approximately 150 mm × 140 mm × 140 mm, comparable to the size of a modern smartphone (Figure 2).

The mechanical layout was optimized to ensure both measurement reproducibility and ease of operation. Dedicated laser mounts enabled manual adjustment of beam position and incidence angle, allowing precise alignment of illumination on the LFA membrane. A fixed camera mount and sample holder maintained a consistent imaging distance and field of view between measurements. The entire enclosure was light-tight, minimizing ambient light interference for both imaging modes.

A removable LFA cartridge slot was added to the front panel, allowing users to easily insert and replace test strips. To isolate the 780 nm wavelength for photothermal measurements, a bandpass filter holder was placed below the camera module. The holder was designed to be easily inserted and removed between imaging modes.

**Figure 3:**
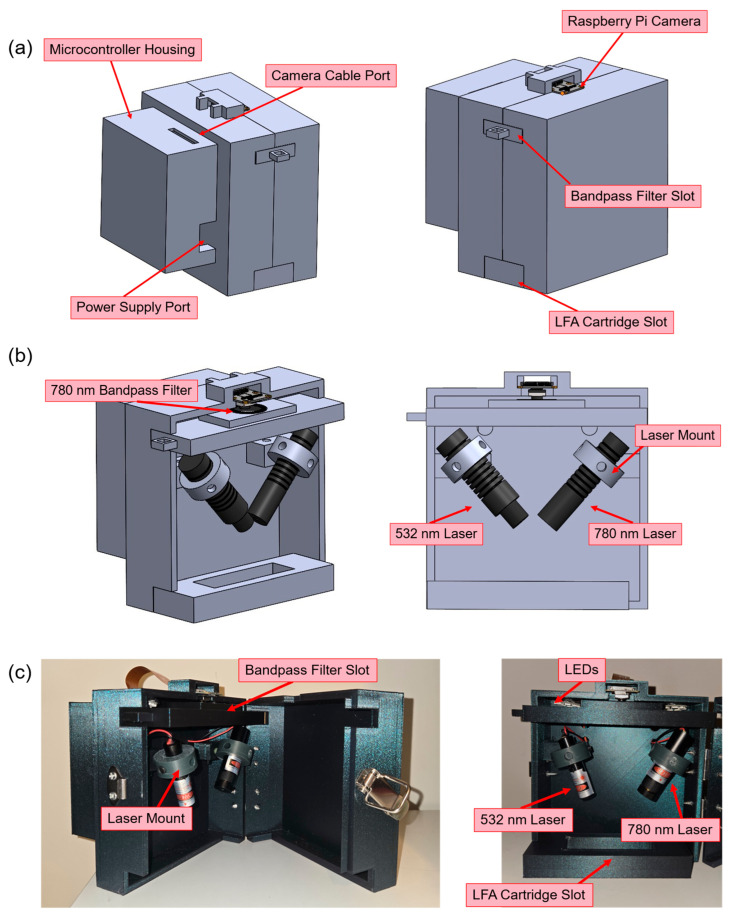
Overview of the portable dual-mode biosensor design: (**a**) CAD model showing external view of the 3D-printed enclosure with labeled access ports and cartridge slot; (**b**) internal optical layout showing the arrangement of optical and electrical components; (**c**) fabricated prototype of the portable biosensor, highlighting the assembled optical and structural components.

The custom PCB and Raspberry Pi 5 were housed in an isolated compartment to reduce thermal and electrical interference. The compartment cover was magnetically attached, providing convenient access for maintenance or component replacement. The finalized system is illustrated in Figure 3, which shows the overall device layout, including the computer-aided design (CAD) model and the fabricated prototype. The total material cost for the complete biosensor system was approximately $500, demonstrating the feasibility of a low-cost, portable diagnostic platform.

### 2.3. Smartphone Integration

A custom smartphone application was developed to provide a user-friendly interface for operating the biosensor and visualizing test results. The application was built in a web-based environment using MIT App Inventor, which hosted an HTML interface with JavaScript and CSS for functionality and styling [31]. The primary purpose of the app was to enable wireless communication with the Raspberry Pi 5, simplifying device control for users.

Communication between the smartphone and Raspberry Pi occurred over a local network using an HTTP-based protocol. Upon establishing a connection, the app transmitted operational commands such as “Start Test” to the Raspberry Pi’s built-in Flask web server [32]. The Raspberry Pi executed the requested operations. All image processing and data analysis were performed locally on the Raspberry Pi to ensure consistent performance independent of smartphone hardware.

**Figure 4:**
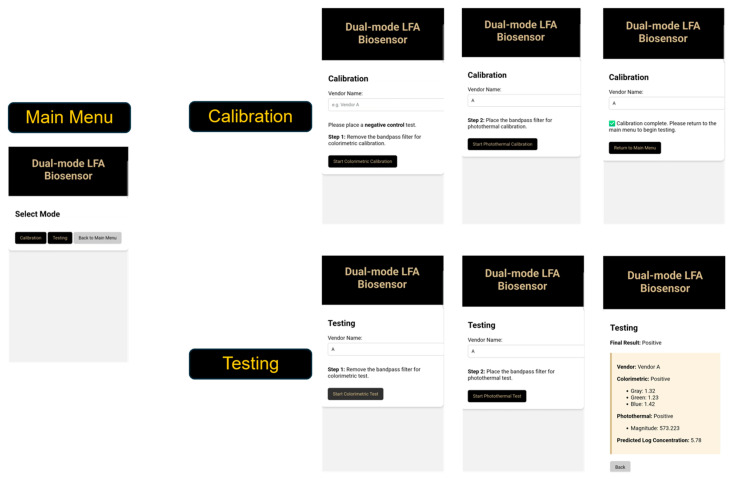
Workflow of the smartphone-integrated biosensor. The smartphone transmits user commands via HTTP to the Raspberry Pi, which controls LED strips, lasers, and camera. Colorimetric or photothermal data are stored locally for processing and results are returned to the smartphone for visualization.

The smartphone served as a graphical control and visualization tool, displaying real-time feedback and final test results (Figure 4). The workflow consisted of two primary modes:

1. Calibration Mode: Captures reference data from negative control LFAs to establish baseline values for each vendor.
2. Testing Mode: Acquires new colorimetric or photothermal data and compares them to stored calibrated reference.

Calibration data were saved locally on the Pi as vendor-specific JSON files, each containing values derived from the control samples. When testing, the system automatically loaded the corresponding calibration file based on the selected vendor. This configuration allowed users to perform calibration once and reuse it across multiple tests without having to manually perform calibration every time for new tests.

### 2.4. Colorimetric Analysis

In the colorimetric mode, the biosensor captures images of lateral flow assays (LFAs) using the Raspberry Pi camera under uniform lighting provided by two circular white LED strips mounted inside the enclosure. The imaging distance was fixed to ensure reproducible measurements.

**Figure 5:**
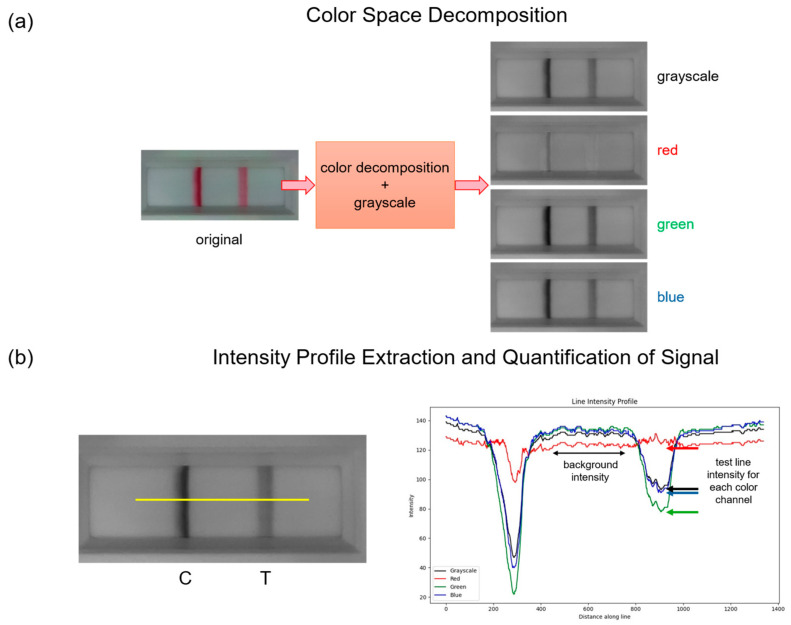
Workflow of colorimetric image processing. (**a**) Capturing a JPEG image of the latera flow assay and decompose into RGB, and grayscale channels. (**b**) The yellow line indicates the path used to extract a line intensity profile across the test line, identifying test line region and background area.

For both calibration and testing, the same acquisition procedure was followed, which is outlined in Figure 5. Once the LFA was inserted into the cartridge slot, the Raspberry Pi captures a single JPEG image that was subsequently decomposed into grayscale and RGB color channels. The red, green, and blue channels were analyzed separately to evaluate which provided the highest contrast between the test line and background.

A line intensity profile was extracted across the test line region using a custom script implemented in Python 3.13.2 with OpenCV and NumPy [33,34]. The test line was identified as a local minimum within the intensity profile, while the background intensity ($I_{\mathrm{background}})$ was determined by averaging pixel values over a selected area adjacent to the test region. The raw line test intensity $\left(I_{\mathrm{test}}\right)$ was normalized to the background using:

(1) $$I_{\mathrm{norm}}=1-\frac{I_{\mathrm{test}}}{I_{\mathrm{background}}}$$

This normalization ensured consistent comparison across independent experiments. The resulting normalized intensity values from the selected color channels were then stored as input features for the machine learning analysis described in Section 2.6.

### 2.5. Photothermal Analysis

The photothermal biosensor used a dual-laser design comprising a 532 nm excitation laser and a 780 nm probe laser. The 532 nm laser was TTL-modulated at 1 Hz (50% duty cycle) through the Raspberry Pi’s GIPO pins to induce periodic plasmonic heating of gold nanoparticles on the LFA membrane. Simultaneously, the continuous 780 nm laser generated a speckle pattern, which was recorded by the Raspberry Pi camera through a 780 nm bandpass filter to isolate the probing wavelength.

For each sample, a total of 150 frames were captured at 30 frames per second. This 5 s duration was selected to capture five full signal cycles, providing sufficient spectral resolution for the 1 Hz target frequency while minimizing data processing time on the selected controller. The region of interest, including the test line, was cropped using a circular mask to minimize background interference and suppress unwanted noise [35,36,37]. Pixels outside the mask were excluded from processing, reducing the contribution of low-contrast boundary regions while improving signal consistency.

The temporal intensity variations in the images were extracted from a speckle-intensity time series. After removing the direct current (DC) offset, a Fast Fourier Transform (FFT) was applied to convert the temporal signal into the frequency domain. The spectral magnitude at the modulation frequency (1 Hz) was identified as the characteristic photothermal response. This magnitude was then averaged across all masked pixels to obtain the representative signal for each test strip.

### 2.6. Machine Learning

Machine learning models were developed to quantitatively relate the optical features extracted from each sensing modality to *Salmonella Typhimurium* concentration. Separate regression models were first trained for the colorimetric and photothermal datasets, followed by a fused analysis to assess the benefit of multimodal integration. Each dataset was divided into training and testing subsets, and all features were standardized prior to model fitting to eliminate scale dependency [38,39,40].

The modeling process involved feature selection, normalization, and regression model training, followed by cross-validation to evaluate prediction accuracy. The trained models were then applied to a blind test dataset to assess generalizability and robustness.

#### 2.6.1. Colorimetric Feature Selection

In the colorimetric mode, input features were extracted from normalized color-channel intensities in the test line area. The green, blue, and grayscale channels were utilized for model training, whereas the red channel was omitted. The red hue of the test line exhibited diminished contrast, rendering it seemingly faded. The ultimate feature set comprised normalized intensities from the green, blue, and grayscale channels, which demonstrated a positive correlation with bacterial concentration. These features indicated the alteration in optical density at the test line.

#### 2.6.2. Photothermal Feature Selection

Photothermal features were designed to capture both temporal and spatial variations in the speckle pattern during periodic plasmonic heating [41,42]. The extracted photothermal features described both temporal fluctuations in the speckle pattern arising from periodic heating and the spatial distribution of the corresponding spectral magnitudes in the frequency domain. Before modeling, the extracted features were standardized and Box–Cox transformed to ensure uniform Gaussian distribution [38,39].

##### Spatial Decorrelation Metrics

Let $I_{t}(x,\;y)$ be the speckle image at time t. For every adjacent pair of frames $\left(I_{t},\;I_{t\;+\;1}\right)$ two measures of frame-to-frame variation were computed and then averaged over the entire temporal sequence:

- Structural Similarity Index (SSIM): Quantifies luminance, contrast, and structural similarity between *I_t_* and *I*_*t*+1_. Higher SSIM values indicate greater similarity and less temporal decorrelation [43].
- Mean Squared Error (MSE): Represents the mean of ${\left[I_{t}\left(x,\;y\right)\;-\;I_{t}\left(x,\;y\right)\right]}^{2}$ over the ROI. Larger MSE reflects larger intensity fluctuations caused by thermally induced phase shifts [44,45].

##### Spectral Distribution Metrics

For each pixel k in the ROI, the temporal intensity $I_{k}(t)$ was Fourier transformed, and the magnitude at the laser modulation frequency ($f_{0}\;=\;1$ Hz) was extracted, yielding one value per pixel. The spatial distributions of these magnitudes were then summarized by their four moments:

- Mean: Average response strength at *f*_0_ across pixels.
- Standard deviation: Spatial uniformity of the response, with a larger value indicating a more heterogeneous distribution.
- Skewness: Asymmetry of the magnitude distribution, reflecting non-uniform regions with disproportionately high or low responses.
- Kurtosis: Tail heaviness or prevalence of outliers within the distribution.

#### 2.6.3. Data Augmentation

To enhance model generalization and robustness against environment variability, data augmentation was applied to both colorimetric and photothermal datasets [46]. These transformations simulated variations that may arise during field operation, such as changes in lighting, camera exposure, or mechanical alignment.

##### Colorimetric Data

Colorimetric images were augmented using photometric transformations that replicated changes in lighting and camera exposure [47]. Three operations were applied to each image.

- Brightness and contrast adjustment: Pixel intensities were randomly scaled and shifted within defined ranges (−25 to 25 for brightness and −30 to 30 for contrast) to reproduce underexposed or overexposed conditions.
- Color temperature modification: RGB channel scaling factors were adjusted to simulate warm or cool lighting conditions, with random values drawn from a range of −20 to 20 relative to predefined reference temperature points.

These transformations created realistic variations in color tone and intensity, helping the model become less sensitive to lighting differences and minor exposure shifts.

##### Photothermal Data

**Figure 6:**
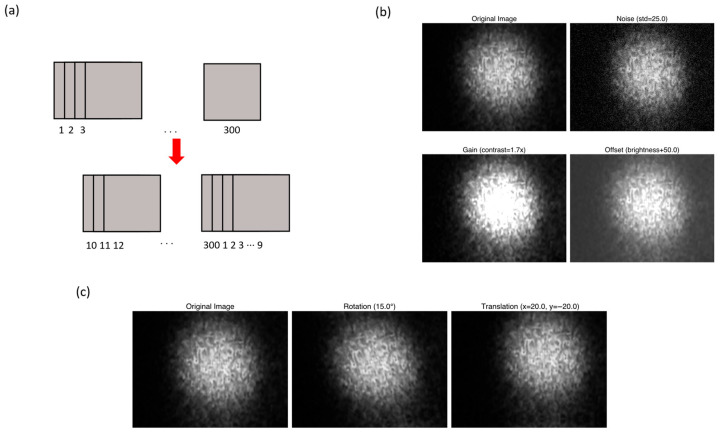
Representative photothermal data augmentation workflow. Extreme parameter values are shown here to help visualize the changes, whereas smaller magnitudes were used in actual model raining to maintain realistic variability. (**a**) Circular time shift simulating temporal phase offset (arrow indicates transformation from original to augmented signal); (**b**) photometric jitter introducing sensor gain, bias, and Gaussian noise variations; (**c**) minor affine transformation applying small rotation and translation to mimic sample misalignment.

Photothermal speckle images were augmented to account for variability in temporal alignment, sensor noise, and sample positioning [48]. Each image sequence was subjected to the following operations (Figure 6):

- Circular time shift: The frame order was rotated by a random integer *k*, effectively changing the starting point of the sequence while preserving temporal continuity. This process corresponds to a phase shift in the frequency domain but leaves the amplitude spectrum unchanged [45,49].
- Photometric jitter: Frame brightness and contrast were perturbed using a random gain (g), bias (b), and Gaussian noise (η). The following operation was conducted to simulate sensor response variations and low-level noise [50].
  (2) $$I_{t}^{’}\left(x,\;y\right)=gI_{t}\left(x,\;y\right)+b+\eta (x,\;y)$$
  where I(x, y) and I’(x, y) denote the original and perturbed pixel intensities, respectively.
- Minor affine transformation: Each frame was randomly rotated and translated to replicate small shifts in sample positioning or camera misalignment. Reflection padding was used to prevent artificial edge effects [44,50,51].

All augmentations were constrained to small magnitudes to preserve the physical characteristics of the speckle pattern while introducing realistic variability.

#### 2.6.4. Model Construction

To quantitatively relate the extracted optical features to *Salmonella* concentration, predictive models were constructed using multivariate linear regression. Previous work with the benchtop system explored three models, including polynomial and logistic regression, to capture nonlinear assay responses. Although those methods provided acceptable fits, the linear regression model was selected for the portable system due to its interpretability, computational simplicity, and robust performance. The linear model can represent the near-linear relationship between optical signals and log-transformed bacterial concentrations, providing a physically interpretable framework for implementation.

##### Model Formulation

The prediction target was defined as the log-transformed bacterial concentration, allowing the model to produce non-negative predictions:

(3) $$y_{log}\;=\log_{10}\left(concentration\right).$$

For a given input feature vector $x=\;[x_{1},\;x_{2},\ldots ,\;x_{p}]$, the general form of the multivariate linear model is expressed as:

(4) $${\hat{y}}_{log}=\beta_{0}+\sum_{i\;=1}^{p}\beta_{i}x_{i},$$

where $\beta_{0}$ represents the intercept, and $\beta_{i}$ denotes the coefficient associated with the $i^{th}$ feature.

Separate models were trained for the colorimetric and photothermal datasets, followed by a fused model that combined the standardized features of both modalities. All models were trained using Python’s scikit-learn library [52].

##### Regularization and Feature Selection

To prevent overfitting and improve model interpretability, Least Absolute Shrinkage and Selection Operator (LASSO) regularization was applied to selected models [53]. The LASSO introduces an $L_{1}$ penalty term to the loss function [29]:

(5) $${\hat{\beta }}^{LASSO}=\arg\underset{\beta_{0},\beta }{\min}\left\{\frac{1}{2n}\sum_{i\;=1}^{n}{\left(y_{i}-\beta_{0}-x_{i}^{T}\beta \right)}^{2}+\lambda {\left\|\beta \right\|}_{1}\right\},$$

where λ controls the penalty strength. The formulation constrains the magnitude of the regression coefficients, driving less-informative features toward zero and achieving implicit feature selection. The optimal value of λ was determined through five-fold cross-validation using the LassoCV function.

For comparison, unregularized linear models were also trained using the same dataset splits and processing steps to evaluate the effect of regularization on prediction performance and generalization.

##### Training and Validation

All features were standardized and Box–Cox transformed prior to model training to reduce skewness. Following the data augmentation procedure described in Section 2.6.3, a total dataset of 15,000 samples was generated to ensure robust model training. These datasets were randomly split into 80% training (12,000 samples) and 20% test sets (3000 samples), with test samples withheld from the training and cross-validation processes. To assess robustness, a bootstrap resampling (1000 iterations) was performed on the training data to estimate confidence intervals for model performance metrics, including the coefficient of determination ($R^{2}$), Akaike information criterion (AIC), and Bayesian information criterion (BIC) [54,55]. The 2.5th and 97.5th percentiles of these distributions were used to compute the 95% confidence intervals for each metric.

### 2.7. Sample Preparation and Testing Procedure

A commercially available *Salmonella* lateral flow assay (STLF-020, BioAssay Works, Imjamsville, MD, USA) was used for all colorimetric and photothermal measurements. The assays are designed for the qualitative detection of broad *Salmonella* species and were selected due to their use of gold nanoparticle tracers.

The bacterial strain for *Salmonella enterica* serovar Typhimurium ATCC 14028 was selected as the testing analyte. Prior to testing, bacterial suspensions were serially diluted in phosphate-buffered saline (PBS) to achieve concentrations ranging from 10^3^ to 10^7^ CFU/mL. All solutions were heat-inactivated at 100 °C for 10 min, to ensure safety. The LFA tests were conducted following the manufacturer’s instructions. For photothermal speckle imaging, test strips were air-dried for approximately 60 min after sample loading to ensure the membrane was dry before measurement, thereby preventing thermal signal loss caused by residual moisture.

For each concentration (10^3^ to 10^7^ CFU/mL) and negative controls, three independent replicate assays were performed to establish the baseline physical dataset. Colorimetric images were captured after reaction was complete, typically 20 to 25 min after loading the sample. The PBS buffer served as the negative control.

#### Blind Testing Procedure

To evaluate the objectivity and generalizability of the models, a blind testing protocol was conducted using a separate set of LFA samples prepared independently by a laboratory technician. The operator responsible for imaging and data acquisition was not informed of the bacterial concentrations or content.

Blind samples included additional assays containing *Escherichia coli*, and *Kocuria rhizophila* to assess model specificity. All samples were randomized before imaging to eliminate potential bias. The blind test data were processed using the trained regression models. Predicted concentrations were then compared against the true values to assess model accuracy user realistic testing conditions.

## 3. Results

### 3.1. Model Performance and Evaluation

Linear regression models were trained and evaluated using datasets derived from each sensing modality to assess predictive performance for *Salmonella* concentration. Separate models were developed with and without LASSO regularization, following the procedures described in Section 2.6.1. The goal was to determine how feature fusion between colorimetric and photothermal signals affected prediction accuracy and robustness.

#### 3.1.1. Linear Regression Models Without Regularization

**Figure 7:**
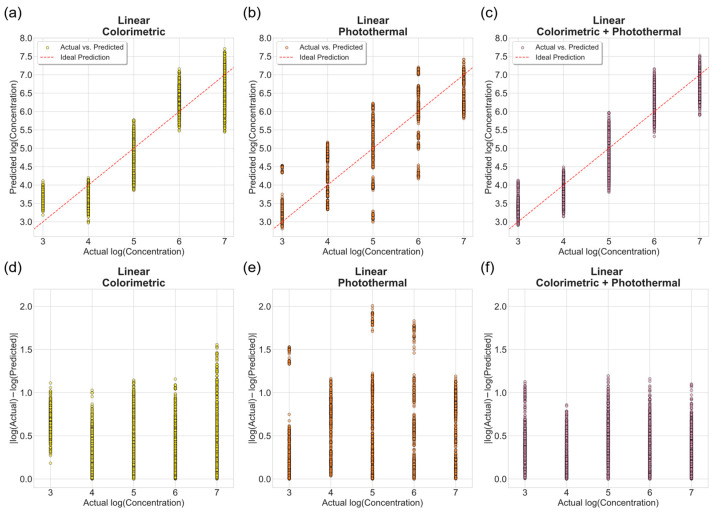
Performance of unregularized linear regression models on the test set (*n* = 3000) for predicting *Salmonella* concentration. (**a**–**c**) Predicted versus actual log concentrations for colorimetric, photothermal, and fused datasets. The dashed red line represents the ideal 1:1 prediction. (**d**–**f**) Corresponding absolute prediction errors expressed as $\left|\log_{10}\left(C_{\mathrm{prediction}}\right)\;-\;\log_{10}\left(C_{\mathrm{true}}\right)\right|$.

Figure 7 shows the predicted versus actual bacterial concentrations obtained from unregularized linear regression models. The colorimetric and fused models exhibited strong linear correspondence with the ideal prediction line, whereas the photothermal model showed larger deviations, particularly at the intermediate range (10^5^–10^6^ CFU/mL), indicating higher variability in the speckle-based features.

Error magnitude plots further highlight this trend. Absolute prediction errors in log_10_ scale were computed as $\left|\log_{10}\left(C_{\mathrm{prediction}}\right)\;-\;\log_{10}\left(C_{\mathrm{true}}\right)\right|$ for each sample. The colorimetric dataset yielded low, consistent errors across all concentrations, whereas the photothermal model showed a broader error distribution due to its higher signal-to-noise ratio. When features from both sensing modes were combined, the fused model achieved the most stable performance, with absolute errors consistently below one log unit for nearly all tests. This result suggests that multimodal feature fusion reduces random noise and compensates for variability present in individual sensing modes.

#### 3.1.2. Linear Regression with LASSO Regularization

**Figure 8:**
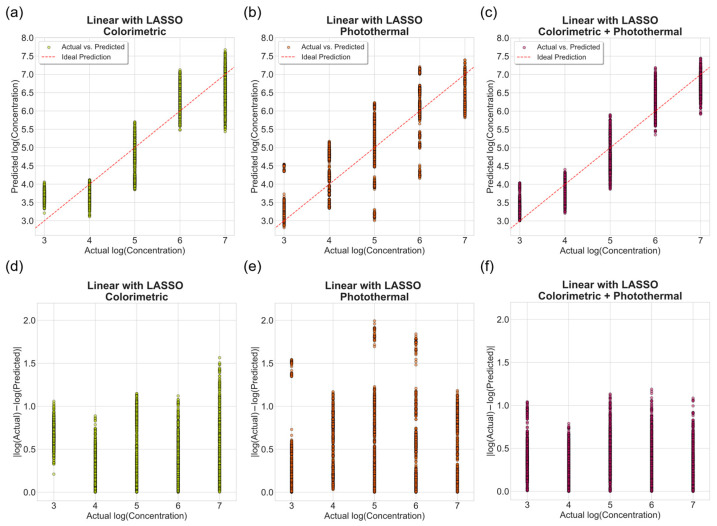
Performance of LASSO-regularized linear regression models on the test set (*n* = 3000) for predicting *Salmonella* concentration. (**a**–**c**) Predicted versus actual log concentrations for colorimetric, photothermal, and fused datasets. The dashed line represents the ideal prediction line. (**d**–**f**) Corresponding absolute prediction errors expressed as $\left|\log_{10}\left(C_{\mathrm{prediction}}\right)\;-\;\log_{10}\left(C_{\mathrm{true}}\right)\right|$.

To evaluate the effect of feature regularization and implicit feature selection, the same datasets were retrained using LASSO-regularized linear regression. The overall predictive trends closely matched those of the unregularized models, indicating that most extracted features contributed meaningfully to the regression performance. As shown in Figure 8, the distribution of absolute prediction errors remained consistent with the unregularized models.

Quantitative performance metrics are summarized in Table 1, including the $R^{2}$, AIC, BIC, and the percentage of predictions within an acceptable error range (±1 log CFU/mL). This range was selected because bacterial concentrations were labeled and modeled in logarithmic form (log_10_ CFU/mL), and deviations within ±1 log unit reflect the scale used for model training and evaluation.

Among the individual sensing modes, the colorimetric model achieved the highest reproducibility ($R^{2}$ = 0.85) with 96% of predictions falling within the acceptable range. The photothermal model, while less accurate ($R^{2}$ = 0.78), maintained reasonable predictive consistency despite inherently higher signal variability. The fused model delivered the best overall performance, achieving $R^{2}$ of 0.91 and nearly 99% or predictions within the acceptable error range, confirming the advantage of combining optical and thermal features for enhanced detection ability. The fused model yielded lower AIC and BIC values despite having more input features, indicating that the improvement in model fit outweighed the complexity penalty imposed by the information criteria.

Regularization produced minimal changes in overall $R^{2}$ values but reduced model complexity by eliminating less informative coefficients. AIC and BIC values remained comparable between regularized and unregularized models, suggesting that LASSO regularization achieved a similar fit quality while simplifying the feature set. Specifically, the LASSO-regularized models retained five out of six features for the photothermal dataset and eight of nine for fused dataset, indicating that most extracted variables carried meaningful predictive value.

### 3.2. Blind Testing and Uncertainty Analysis

To evaluate the real-world performance of the trained regression models, blind testing was conducted using eight unknown samples containing varying combinations of *Salmonella Typhimurium*, *Escherichia coli*, and *Kocuria rhizophila* (Table 2). The models described in Section 2.6.4 were used to predict *Salmonella* concentration for each sample, allowing assessment of model accuracy, specificity, and cross-pathogen robustness.

#### 3.2.1. Prediction of Blind Test Samples

Table 3 summarizes the predicted and actual *Salmonella* concentrations for all blind test samples. The results demonstrate that colorimetric and fused models reliably predicted *Salmonella* concentrations down to 10^4^ CFU/mL. The photothermal model exhibited greater deviation, particularly at mid-range concentrations. The fused model produces the most consistent and accurate predictions across the full concentration range, confirming that integrating optical and photothermal features improves robustness and overall detection reliability. However, samples containing only non-target bacteria (*E. coli* or *K. rhizophila*) were not classified as negatives, as all models returned low but non-zero concentration estimates of approximately 10^3^ CFU/mL. This outcome suggests that, at the current sensitivity level, the system cannot yet reliably distinguish between *Salmonella* presence and background signal near the detection limit.

#### 3.2.2. Model Comparison

**Figure 9:**
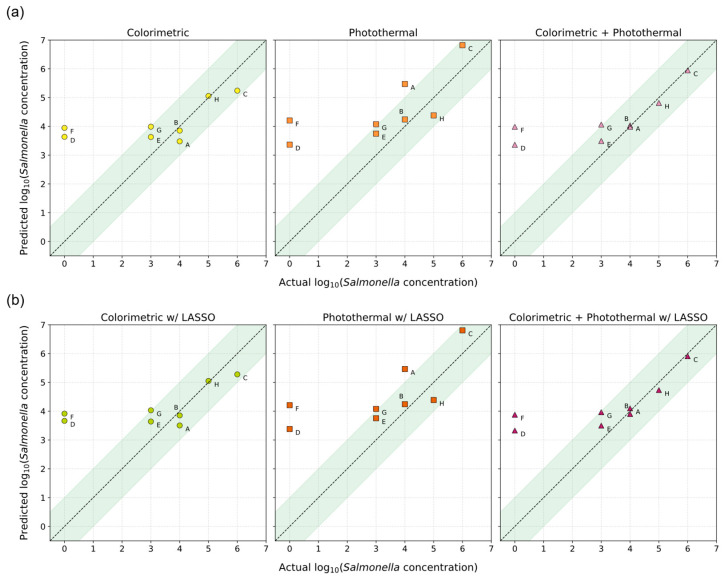
Predicted versus actual *Salmonella* concentrations for blind test samples. (**a**) Unregularized linear regression models using colorimetric (yellow circles), photothermal (orange squares), and fused (purple triangles) datasets. (**b**) Corresponding LASSO-regularized models. Labels A-H correspond to the specific blind test sample listed in Table 2. The dashed line represents ideal prediction, and the shaded green region indicates the ±1 log error range.

Figure 9 illustrates the predicted versus actual *Salmonella* concentrations for all blind test samples, with shaded green bands representing the ±1 log acceptable error range. Predictions closer to the ideal diagonal line indicate higher accuracy. Both the colorimetric and fused models produced results that largely fell within the acceptable range, whereas the photothermal model showed greater dispersion.

Applying LASSO regularization had minimal impact on overall prediction accuracy, as shown in Figure 9b. The near-identical performance of regularized and unregularized models suggests that the extracted features were not redundant and that each contributed meaningfully to the regression outcome. While fusion of optical and thermal features improved consistency and reduced error variance, none of the models successfully classified negative or non-target samples as true negatives. All produced non-zero outputs, reinforcing that the current detection threshold lies between negative and 10^4^ CFU/mL.

#### 3.2.3. Uncertainty Quantification

To assess prediction reliability and model robustness, uncertainty was quantified using a bootstrap-based confidence interval approach. For each sensing modality, the training dataset was resampled 1000 times with replacement, and a new regression model was refitted at each iteration Predicted concentrations for each blind test sample were then recorded across all bootstrap iterations. The 2.5th and 97.5th percentiles of the resulting distributions defined the lower and upper bounds of the 95% confidence interval in log space. The relative uncertainty of each sample was then calculated as

(6) $$Percent\;uncertainty=\left(10^{\frac{{log(CI}_{\mathrm{upper}})-{log(CI}_{\mathrm{lower}})}{2}}-1\right)\times 100\%.$$

The calculated uncertainties, which serve as a measure of assay reproducibility (analogous to the coefficient of variation), ranged between 4% and 13% across all models. The colorimetric model exhibited the lowest uncertainty (4–6%) while the photothermal and fused models exhibited slightly higher uncertainty (7–13%) due to greater temporal variability in speckle-derived measurements and higher feature dimensionality.

### 3.3. Summary of System Performance and Limit of Detection

The overall performance of the developed biosensor was evaluated by comparing the results from visual inspection, the previous benchtop system, and the final portable dual-mode platform. The limit of detection was defined as the lowest bacterial concentration that could be reliably distinguished from the negative control.

For the visual colorimetric assay, *Salmonella* test line became indistinguishable to the naked eye at concentrations below 10^5^ CFU/mL, corresponding to a practical LOD of approximately 10^5^ CFU/mL. Similarly, both individual sensing modes of the benchtop system yielded an LOD near 10^5^ CFU/mL.

In contrast, the portable dual-mode biosensor demonstrated improved sensitivity. Using machine learning regression models trained on fused colorimetric and photothermal features, the system consistently differentiated 10^4^ CFU/mL samples from PBS and non-*Salmonella* controls. Although detection at 10^3^ CFU/mL remained inconsistent, the portable device maintained stable quantitative predictions at 10^4^ CFU/mL, whereas visual and benchtop analysis failed.

This improvement represents an order-of-magnitude increase in detection capability, from a limit of 10^5^ CFU/mL observed in both visual inspection and our previous benchtop study to 10^4^ CFU/mL with the portable dual-mode system. The observed gain in sensitivity can be attributed to improved optical optimization in the compact device design, robust data fusion of colorimetric and photothermal features, and machine learning-based regression that reduced the influence of user subjectivity and measurement variability. Overall, these findings confirm that the integrated dual-mode machine learning-assisted biosensor enables more quantitative, low-cost, and field-deployable detection of foodborne pathogens.

## 4. Discussion

This study presents the development of a smartphone-integrated, dual-mode biosensor that combines colorimetric and photothermal speckle imaging to enhance the sensitivity of lateral flow assays. Through regression modeling, the portable system achieved an order-of-magnitude improvement in detection sensitivity compared to visual interpretation and benchtop analysis, reducing the limit of detection from 10^5^ CFU/mL to 10^4^ CFU/mL, as reported in our previous work. The device maintained consistent performance during blind testing, demonstrating its potential for quantitative and field-deployable pathogen detection using standard smartphones.

While the colorimetric sensing mode alone provided high reproducibility and stability, integrating photothermal speckle imaging contributed robustness, compensating for signal variability that can arise under different lighting or surface conditions. The fusion mechanism relies on the complementary nature of two signals. The colorimetric mode provides a low-variance baseline that stabilizes the prediction, while the photothermal mode contributes high-sensitivity features that persist even when the visual test line fades. Mathematically, the regression model leverages the colorimetric stability to constrain the higher variance inherent in the speckle fluctuations, preventing noise from driving false positives while retaining the sensitivity benefits of the thermal signal. Although the improvement in prediction accuracy from multimodal fusion was moderate under current testing conditions, the dual-mode framework may become more impactful under variable environmental or sample conditions.

Several factors currently limit the system’s detection sensitivity and practical implementation. The most notable challenge is the difficulty in differentiating between negative controls and 10^3^ CFU/mL samples. This may be influenced by residual membrane moisture, which can reduce signal strength during photothermal imaging. In addition, timing differences between colorimetric and photothermal measurements can introduce variability. Since colorimetric imaging is performed immediately after the readout period specified by the vendor, while photothermal imaging requires a drying step, the delay may lead to test line bleeding. To ensure real-world applicability, the proposed system design can be upgraded to include a low-power heating element or fan to accelerate membrane drying after sample application. This would synchronize the optimal signal reading times for both modalities, minimizing the delay and preventing signal degradation caused by residual moisture. Future work will focus on several directions:

1. Robustness: Test additional LFA vendors to assess reproducibility and model generalizability
2. Sensitivity: Explore higher-power excitation, improved lighting, and alternative image sensors to achieve lower detection limits
3. Workflow: Use controlled drying to reduce timing disparities and prevent test line bleeding.
4. Application generalization: Adapt the platform to multiplexed or non-pathogen assays to expand applicability to clinical, food safety, and environmental diagnostics.

Overall, this study established a foundation for quantitative, low-cost, and field-deployable biosensing that bridges optical sensing and computational power. The integration of machine learning with dual optical modalities is a step toward robust, accessible diagnostic tools capable of providing reliable results outside traditional laboratory settings.

## Figures and Tables

**Table 1:** Performance summary of unregularized and LASSO-regularized linear regression models trained on colorimetric, photothermal, and fused datasets. Metrics include the coefficient of determination ($R^{2}$), Akaike information criterion (AIC), Bayesian information criterion (BIC), and the percentage of predictions within ±1 log CFU/mL error range. Values in parentheses indicate 95% confidence intervals obtained from 1000 bootstrap iterations.

Trained Dataset	Regularized	*R* ^2^	AIC	BIC	Predictions Within Acceptable Error Range
Colorimetric	No	0.849 (0.848, 0.851)	4926.9 (4896.0, 4958.7)	4957.0 (4926.0, 4988.7)	96.4%
	Yes	0.849 (0.847, 0.850)	4942.0 (4906.9, 4977.0)	4971.9 (4937.0, 5007.0)	95.9%
Photothermal	No	0.778 (0.777, 0.778)	6093.1 (6084.8, 6102.0)	6123.1 (6114.8, 6132.0)	88.3%
	Yes	0.778 (0.777, 0.778)	6093.5 (6805.4, 6102.4)	6123.6 (6115.4, 6132.4)	88.3%
Colorimetric + Photothermal	No	0.910 (0.905, 0.915)	3425.7 (3405.3, 3448.0)	3455.7 (3435.4, 3478.0)	98.7%
	Yes	0.908 (0.907, 0.908)	3460.0 (3434.2, 3485.8)	3490.0 (3464.2, 3515.8)	95.9%

**Table 2:** Composition of blind test samples used for model validation. Each sample contained varying combinations of *Salmonella Typhimurium*, *Escherichia coli*, and *Kocuria rhizophila*. The actual log bacterial concentrations represent the total bacterial load, while the actual log *Salmonella* concentration indicates only the portion attributable specifically to *Salmonella* within each sample.

Test	Bacteria	bacterial Concentration (CFU/mL)	*Salmonella* Concentration (CFU/mL)
A	*Salmonella*	4	4
B	*Salmonella + E. coli*	4	4
C	*Salmonella*	6	6
D	*Kocuria rhizophila*	5	0
E	*Salmonella + E. coli*	3	3
F	*E. coli*	5	0
G	*Salmonella*	3	3
H	*Salmonella + E. coli*	5	5

**Table 3:** Predicted *Salmonella* concentrations for blind test samples obtained using colorimetric, photothermal, and fused regression models, with and without LASSO regularization. Each value represents the predicted log concentration generated by the trained models described in Section 2.6.4.

	Predicted Log (*Salmonella* Concentration-CFU/mL)					
*Salmonella* Concentration (CFU/mL)	Colorimetric	Photothermal	Fused	Colorimetric w/LASSO	Photothermal w/LASSO	Fused w/LASSO
4	3.48	5.47	4.04	3.50	5.46	4.10
4	3.85	4.24	3.99	3.85	4.24	3.90
6	5.24	6.82	5.95	5.28	6.81	5.91
0	3.64	3.37	3.36	3.66	3.38	3.33
3	3.63	3.75	3.49	3.64	3.76	3.50
0	3.95	4.20	3.99	3.91	4.21	3.88
3	3.99	4.08	4.06	4.03	4.08	3.97
5	5.06	4.38	4.82	5.05	4.39	4.73

## Data Availability

All data and code supporting the findings of this study are publicly available in the following GitHub repository (Version v1.0.0): https://github.com/jully-blackshare/MDPI_Dual_Mode_Biosensor (accessed on 2 December 2025).
